# The Complete Diagnosis of Disease of the Heart

**Published:** 1896-10-24

**Authors:** J. Mitchell Bruce

**Affiliations:** Physician to the Charing Cross Hospital and to the Hospital for Consumption, Brompton; Examiner in Medicine Royal College of Physicians, London.


### Medical Progress and Hospital Clinics.

[*The Editor will be glad to receive offers of co-operation and contributions from members of the profession. All letters should be addressed to* The Editor, at the Office, 28 & 29, Southampton Street, Strand, London, W.C.]

### THE COMPLETE DIAGNOSIS OF DISEASE OF THE HEART.

By J. Mitchell Bruce, M.D., F.R.C.P., Physician to the Charing Cross Hospital and to the Hospital for Consumption, Brompton; Examiner in Medicine Royal College of Physicians, London.*

The first step in the complete diagnose of disease of the heart is its location—*i.e.*, whether it is valvular or non-valvular; and, if valvular, which of the valves it involves, and in which direction its function is affected.

The second step is one which is too often neglected, viz., the determination of the nature and kind of the disease of the valve. "Valvular disease" is a mere anatomical expression, and "mitral obstruction" is but little better unless the nature of the lesion be set forth —*i.e.*, whether it arise from rheumatism, chorea, septicæmia, syphilis, gout, degeneration from disordered nutrition, or strain—whether sudden or protracted, including in the latter term Bright's disease and prolonged cardiac excitement, as in Graves's disease, and perhaps others.

As showing the importance of deciding on the nature of the disease by which the valves are affected two kinds of valvular lesion may be contrasted—a lesion of the aortic valves consequent on rheumatic endocarditis, and one of syphilitic origin. Is it of any genuine importance to differentiate these? So important is it that an opinion on such a case is valueless until it is done. The first case may live indefinitely, because the lesion is the scar of an old endocarditis, which, however, may recur and set up fresh mischief; while the second case will quite probably die shortly, and perhaps suddenly, because the syphilitic disease has its seat in the *vasa vasorum* of the aortic wall, and involves the coronary arteries of the heart. In regard to treatment also this question of "kind" is of great importance. The old rheumatic lesion calls for no anti-rheumatic treatment, but the active syphilitic disease demands immediate treatment by large doses of iodides,

The third step in the complete diagnosis of disease of the heart is to determine the presence, character, and completeness of compensation.

The fourth step is as to the existence of failure of compensation; and, finally, we have to consider the cause of this failure. I would insist on the importance of the last point, a matter which is constantly being neglected. We ought never to be content with what is usually regarded as the end of diagnosis, *i.e.*, the discovery of failure of compensation. We must get at the cause which is at work if failure have occurred. Over-work, insufficient exercise, under-feeding, over-feeding, cardiac poisons, mental distress or strain, anæmia from hæmorrhage, insanitary modes of life or work, uterine disorders, affections of the lungs taxing the right heart, affections of the vessels taxing the left heart, including Bright's disease—these are examples of the various causes at work in the production of cardiac failure, and both the prognosis and the treatment of the failure depend on the ascertainment of its cause.

In our study of heart disease two points always stand prominently forward; first, the cardiac work, or the energy demanded of the heart: second, the energy supplied in latent form to the heart by the blood, which in turn is derived from the food. These factors are the keys to the prognosis and treatment of heart disease. They must be ascertained in each case, and kept before the mind in practice.

The cardinal condition of efficient action of the heart is that the driving power shall be greater than the resistance ahead. Unfortunately this is not always the case.

1. The work may be excessive even for the healthy heart, and *à fortiori* for the diseased or hypertrophied one. This is "strain" or "overwork," as it is daily seen in the poor man, in whose case mere rest in hospital is often of itself sufficient treatment.

2. The work may be insufficient, and the latent energy of the food may accumulate in the myocardium in the form of fat. This is the degenerative heart of the well-to-do, which exercises—walking or otherwise, formal and graduated, as taught in this country for the last fifty years by Stokes, Quain, &c.—do so much to remedy.

3. Nutrition may be excessive, as in the indolent overfed—a condition to be treated by regulation of the diet, combined with graduated exercises.

4. Nutrition may be defective from various causes—disease of the coronary arteries; general anæmia, whether this be caused by hæmorrhage, exhausting disease, or want of food; insanitation; or toxæmia, from excess in tea, tobacco, alcohol, &c.

In any case, however, the first and most important thing to do is to discover the cause of the want of balance between the driving power of the heart and the work which has to be done.

* Abstract of a lecture delivered October 15th, 1896.

